# Temperature-Dependent Phase Evolution in FePt-Based Nanocomposite Multiple-Phased Magnetic Alloys

**DOI:** 10.3390/nano12234122

**Published:** 2022-11-22

**Authors:** Ovidiu Crisan, Alina Daniela Crisan, Nirina Randrianantoandro

**Affiliations:** 1 National Institute for Materials Physics, P.O. Box MG-7, 077125 Magurele, Romania; 2 Institut des Molécules et Matériaux du Mans, UMR CNRS 6283, Faculté des Sciences & Techniques, Université du Maine Avenue Olivier Messiaen, CEDEX 09, 72085 Le Mans, France

**Keywords:** magnetic multiphase materials, ^57^Fe Mössbauer spectroscopy, melt spun ribbons

## Abstract

A quaternary Fe–Pt–Nb–B alloy has been fabricated by the melt spinning method with the purpose of the formation of crystallographically coherent multiple magnetic phases, emerging from the same metastable precursor, as well as to investigate the phase interactions and the influence of their coupling on magnetic performances. For this purpose, extended structural and magnetic investigations were undertaken by making use of X-ray diffraction, transmission electron microscopy, and ^57^Fe Mössbauer spectroscopy, as well as magnetic measurements using SQUID magnetometry. It was documented that intermediate metastable phases formed during primary crystallization, in intermediate stages of annealing, and a growth-dominated mode was encountered for the secondary crystallization stage upon annealing at 700 °C and 800 °C where fcc Fe3Pt and fct Fe2B polycrystalline were formed. The Mössbauer investigations have documented rigorously the hyperfine parameters of each of the observed phases. The fcc A1 FePt phase was shown to exhibit a peculiar ferromagnetic transition, and this transition has been proven to occur gradually between 300 K and 77 K. The magnetic measurements allowed us to identify the annealing at 700 °C as optimal for obtaining good magnetic features. Coercive field dependence shows similarities to the random anisotropy model for samples annealed at 500 °C to 700 °C which are nanocrystalline. These results show good perspectives for use in applications where different magnetic states are required at different operating temperatures.

## 1. Introduction

Recent developments in the field of renewable energies, autonomous vehicles, smart buildings, and other IoT household applications have seen an emerging need for novel, reliable magnets, and magnetic sensing, with on-chip integration capabilities. The magnetic multiphase materials, derived from the Fe–Pt systems, have been under scrutiny for several years now, due to their good performance given by the combined effect of ordered microstructure and magnetic coupling between the different magnetic phases. In some applications, such as sensor integration into logic devices [1], FePt-based magnets have proven more reliable than classic Nd–Fe–B magnets in obtaining a defined magnetic state, depending on the needed operating temperature. Much research effort has been spent on developing nanocomposite FePt-based magnets that are highly corrosion-resistant and with a working temperature which is higher than the traditional Nd–Fe–B ones [2,3,4,5].

FePt usually crystallizes in a disordered face-centered-cubic fcc A1 structure which upon annealing, for quasi-equiatomic stoichiometry, transforms into an ordered face-centered-tetragonal fct phase with high coercivity and large magnetocrystalline anisotropy. It has been shown [2] that by suitable additions to the initial chemical composition, the FePt-based alloys may achieve a state where several magnetic phases co-exist and the overall magnetic behavior of the alloy can benefit from the interplay between the performances of the individual magnetic phases, powered by the magnetic coupling between phases. Such interplay is greatly facilitated if conditions of crystallographic coherence of the constituent phases are met. This means that in order to make the coupling more efficient, the two phases shall emerge via crystallization processes from the same amorphous-like, or metal–solid solution, precursor. Moreover, by finely tuning synthesis parameters such as composition, stoichiometry, or post-synthesis annealing parameters, a suitable arrangement of magnetically coupled nanograins of the different magnetic phases may be achieved. This in turn facilitates obtaining enhanced magnetic performances due to the coupling-driven interplay of the different magnetic phases. One example of this kind of multiphase material is the classic Finemet-type FeCuNbSiB alloy where very good magnetic properties are achieved due to the interplay between the Fe_3_Si nanograins and the soft magnetic amorphous residual matrix Fe–Nb–B [3]. Another example is represented by the hard-soft nanocomposite magnets FePt/Fe_3_B, [4] where the overall behavior of the alloy benefit from the high magnetization of the Fe_3_B and the high coercivity of the FePt, thus giving an energy product that is superior to that of the two constituent magnetic phases.

The magnetic alloy that we present in this paper, the FePtNbB system, emerged from the lessons learned from the two examples noted above. Starting from the FePt system, we have added Nb and B, in an attempt to recreate a product similar to the Finemet alloy, an initially amorphous precursor. From this amorphous precursor, upon appropriate annealing, the aim is to crystallize two different magnetic phases, the FePt and the Fe boride, and to investigate the coupling-mediated interplay between these two phases. One of the suitable ways to synthesize such magnets is to use the melt spinning technique for casting amorphous-like ribbons from which, after suitable annealing procedures, the desired microstructure of nanosized grains emerging from the same metastable precursor may be achieved. Inspired by the well-known example of the Finemet alloy [3] where glass-forming elements such as B and grain growth suppressor elements such as Nb are added to a classical Fe3Si-based soft magnet, in the FePt alloy, B and Nb can also be added in order to form in the as-cast state an initial amorphous-like precursor.

In the field of multiphase magnets, large efforts have been dedicated to optimization of the microstructure and to obtain suitable grain arrangements for an effective coupling, capable of ensuring enhanced magnetic properties. For instance, in FePt (B–Ag) granular films, Tsai et al. [5] have shown that after annealing, the microstructure contains intertwined FePt grains and immiscible (B, Ag) phases, the process occurring through diffusion of high mobility (B, Ag) atoms within FePt grain boundaries, facilitating thus better magnetic performances. In multiphase Fe-B/FePt-type nanocomposite ribbons, Chang et al. [6] have compared the coercivities of binary FePt with the similar property of a nanocomposite FePt with addition of 18 at% B. It has there been shown that while in the binary FePt, the coercivity barely raised to 2 kOe, but in the similar alloy but with B addition, the formation of iron borides upon annealing as additional magnetic phases led to an enhancement not only of the coercivity, but also of the energy product. This has been attributed [6] to the strong exchange coupling between the grains of different magnetic phases, in this instance the FePt and the Fe borides Fe_2_B and Fe_3_B. In the case of FePt/Fe multiphase nanocomposite films, Gayen et al. [7] have shown that the introduction of a buffer carbon layer between the two magnetically different thin films, FePt and Fe, effectively promotes a different kind of magnetic coupling, the interlayer exchange coupling which depends strongly on interlayer materials and interface morphology and causes enhancement of the saturation magnetization of the nanocomposite. Nevertheless, for an effective exchange coupling between phases it has been shown that the two different magnetic phases should emerge from a common amorphous-like precursor, should be crystallographically coherent, and the grain arrangement has to be controlled at the nanometer scale.

There have been reports on the preparation and characterization of quaternary alloys Fe–Pt–M–B, where M = Zr, Nb, and Ti. Makino et al. [8,9,10] studied the (Fe_0.55_Pt_0.45_)–M–B alloys formed by rapid quenching and the quaternary Fe-Pt-Zr-B alloy with equiatomic Fe/Pt ratio. For the (Fe_0.50_Pt_0.50_)_78_Zr_4_B_18_ alloy they obtained the best magnetic properties, i.e., a coercivity value of Hc = 688 kA/m. Nevertheless, no structural characterization was made in the study reported in [8], in order to explain the origin of the increased coercivity, and no exchange spring mechanisms between the constituent phases have been proved. In our previous works [2], we have shown that in Fe-rich Fe–Pt–Nb–B alloys the microstructure is highly sensitive to the stoichiometry of the as-cast alloy. A boron content of about 8–9% is proven to be not enough to ensure an amorphous-like as-cast state and the samples is obtained in its crystalline A1 structure, therefore higher boron content of about 18–20% is needed for obtaining a disordered, amorphous-like structure in the as-cast state.

The matter of temperature-dependent magnetic ordering in the FePt and FePt-based alloys, films, and nanoparticles is still a hot topic, since the material is widely investigated due to its applicability in magnetic recording, as proven by several, very recent, works. The investigation of the magnetocrystalline anisotropy energy dependence on the magnetic moment was investigated in an FePt system by DFT calculations [11] and the temperature-dependent magnetic ordering has also been studied in several recent works [12]. The anisotropy field of L1_0_ FePt near the Curie temperature (T_C_), recently performed [13], was shown to also be composition-dependent. Mechanisms of magnetic switching were investigated for L1_0_ FePt in [14] while the effects of Fe amount in FePt films [15] were shown to strongly influence the magnetic ordering.

The present work is dedicated to the detailed structural and morphological study of the magnetic properties of the multiple phased Fe–Pt–Nb–B melt spun alloy. Its phase evolution during annealing and coupling-induced interplay between various magnetic phases will be thoroughly investigated via Mössbauer spectrometry and magnetic investigations. In one of the constituent FePt phases of the alloy, a paramagnetic to ferromagnetic transition is documented below 300 K and this magnetic ordering is shown to be temperature-dependent.

## 2. Materials and Methods

### 2.1. Synthesis

For the proposed study, the synthesis of the alloy has employed an out-of-equilibrium technique involving the ultra-rapid solidification from the melt–melt spun technique. To establish the needed stoichiometry, a compositional strategy was established which takes into account previous knowledge acquired in such systems: the need to have enough boron content to ensure an amorphous-like initial state and the need to have a few percent of a refractory element, such as Nb atoms, playing the role of nucleation centers for the incipient formation of crystallites during the primary crystallization of the alloy during annealing. With all this in mind, we have established the alloy’s nominal composition to be Fe_68_Pt_13_Nb_2_B_17_ in atomic percent. The alloy was synthesized using the abovementioned melt spun technique. The first step was to mix together the needed quantities of the elements, as powders and flakes of high purity. All materials were of at least 99.9% purity and were purchased from Alfa Aesar GmbH, Karlshruhe, Germany. They were afterwards melted together in a home-made induction furnace with rigorously controlled parameters, up to a melting temperature of 1300 °C. The primary alloy was 3 times re-melted to prevent element segregation and to improve its chemical homogeneity. A total amount of 5 g was used for each sample. The rapid solidification of the melt was performed on a Bühler Melt Spinner SC, from Edmund Bühler GmbH, Bodelshausen, Germany, with a protective Ar atmosphere. The obtained melt was purged onto the surface of a Cu wheel. The wheel was 40 cm in diameter and rotated with 2000 rot/min. The melt was purged from the quartz tube through a circular nozzle using an Ar gas pressure of circa 40 kPa, or 400 mbar. The melt solidified ultrafast with a cooling rate of about 10^6^ K/min and the rotating wheel cast away long and continuous ribbons, 30 microns thick and 2–3 mm wide.

For achieving full crystallization of the ribbons, as well as occurrence of the desired magnetic phases, the as-obtained ribbons were subsequently annealed via an isothermal procedure. The annealing temperatures chosen were 500 °C, 600 °C, 700 °C, and 800 °C. During the isothermal annealing, the heating rate was stabilized at 5 K/min and annealing time (or the time spent at maximum temperature) was set to 20 min. The annealing procedure was conducted in high vacuum conditions (10^−5^ mbar) to avoid oxidation during annealing.

### 2.2. Characterization

Several investigation techniques were used to study the structural and morphological effects on the alloy microstructure, its changes induced by annealing as well as the phase evolution with the annealing temperature. In addition, a thorough study of magnetic properties in various stages of annealing as a function of applied field or as a function of temperature were performed. These studies were carried out using X-ray diffraction (XRD), transmission electron microscopy (TEM), ^57^Fe Mössbauer spectrometry (MS), and magnetic characterizations. For XRD, a Bruker D8 Advance (from Bruker AXS GmbH, Karlsruhe, Germany) using the Cu K_α_ radiation wavelength of 1.54 Å was used. The geometry used for acquiring the XRD was the classical θ–2θ with an incidence angle of 1.5° in an angular interval 20° to 90°. From XRD data, by using full profile analysis, lattice parameters and average grain sizes were obtained. The software used for the full profile analysis was MAUD (Materials Analysis Using Diffraction software version 2.99, University of Trento, Italy).

The ^57^Fe Mössbauer spectrometry is performed in transmission geometry at 300 K and 77 K using a Mössbauer bath cryostat from Oxford Instruments (Abingdon-on-Thames, Oxfordshire, UK) with a ^57^Co source in a Rh matrix. For the TEM imaging, a JEOL JEM 2100 electron microscope with a 200 kV acceleration voltage and 2 nm beam resolution (from JEOL (UK) Ltd., Hertfordshire, UK) was used. The samples to image were thinned by reactive ion etching using a plasma discharge technique. Magnetic characterization was performed with the SQUID (superconducting quantum interference device) unit of a MPMS (magnetic properties measurement system) from Quantum Design (Quantum Design Europe GmbH, Darmstadt, Germany), under an applied field of up to 5.5 Tesla, in parallel and perpendicular geometry and temperatures ranging from 4.2 K to 300 K.

## 3. Results and Discussion

### 3.1. Structural Investigations

**Figure 1:**
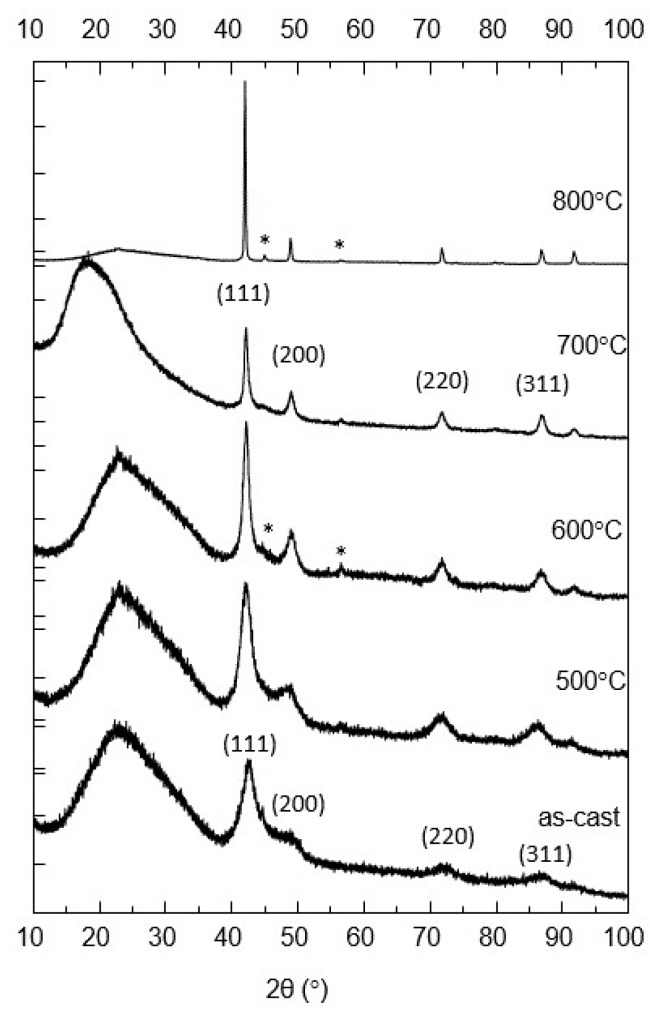
XRD patterns of the samples as-cast and annealed at 500°C to 800°C.

The ribbons, either in their as-cast state or annealed, were investigated using XRD and Mössbauer spectrometry. Figure 1 shows the X-ray diffractograms of the as-cast and annealed samples.

The diffractogram of the as-cast sample consists of several well-formed, very broad Bragg lines. These broad lines have been indexed as belonging to the face-centered cubic (fcc) A1 phase, their (hkl) assignation being shown on the graph. As proven by the lines broadening, the A1 fcc structure is quite disordered.

In order to correctly assess the observed Bragg reflections to the particular (hkl) planes of the A1 structure, full-profile analysis of the XRD patterns has been carried out using MAUD software (version 2.99, University of Trento, Italy). Bragg lines corresponding to the main reflections of the fcc phase were unambiguously identified thus proving that the structure of the as-cast sample has disordered fcc A1 symmetry. Based on the results of the MAUD fitting we have calculated, using the integral breadth approach [16,17,18], the crystallite size for all the Bragg peaks indexed in the fcc A1 system. Using the lattice parameters, FWHM, and mixing parameters of each Bragg reflection, the integral breadth method calculated the average crystallographically coherent domain size associated with the average size of the grains. Based on this approach, the mean grain size for the as-cast sample was found to be 3 ± 1 nm.

All the calculated lattice parameters as well as the average grain sizes for the as-cast and annealed samples are given in Table 1.

For the sample annealed at 500 °C, the lines were narrower, the (200) reflection was better formed than in the case of the as-cast sample, but no additional Bragg peaks were observed. The broad line centered at around 25° is due to the glass sample holder and the glue used to hold the ribbons during measurements. This broad feature was preserved at all annealing temperatures, until 800 °C, where the intensity of the main Bragg peak was very high and the broad feature appears strongly diminished due to scaling. At 500 °C, the same A1 fcc broad line pattern was observed but line profiles were better formed with smaller linewidth than in the as-cast state.

At 600 °C the main lines of the fcc Fe_3_Pt phase were observed and the process of narrowing continued further, as expected. All the identified (*hkl*) reflections are shown on the graph. Starting at 600 °C, a small Bragg peak, attributed to the main Bragg reflection of the Fe_2_B tetragonal phase, began to be observed.

At 700 °C the lines of the fcc phase (the same fcc Fe_3_Pt and Fe_2_B phases being indexed in the XRD diagram) were even narrower, however, the intensities of the peaks attributed to the Fe_2_B phase were very weak. The XRD diagram at 800 °C shows a stronger narrowing of the Bragg reflections, corresponding to a sharp increase of the average grain size. The sample was completely crystallized and the fcc Fe_3_Pt and Fe_2_B are the only phases visible in the diagram.

**Figure 2:**
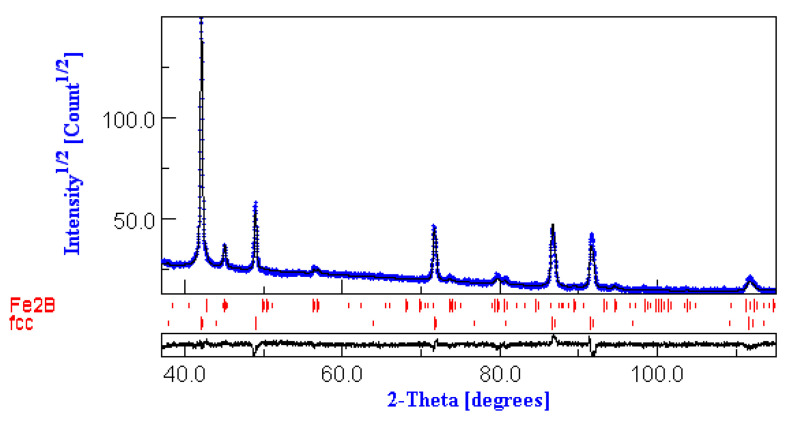
MAUD fitting of the XRD diagram of the sample annealed at 800 °C.

To prove the co-existence of the fcc Fe_3_Pt and the fct Fe_2_B in the sample annealed at 8000 °C and also to exemplify the goodness of the fit with the MAUD software, the fitting of the sample annealed at 800 °C is shown in Figure 2. It can be seen that the experimental data in this diffractogram fits very well with the full-profile refinement fitting model. The analysis of the evolution of lattice parameters of the indexed phases and the average grain sizes derived from the fit of the XRD spectra, given in Table 1, provide interesting insight into the phase evolution with annealing temperature.

Firstly, it is worth mentioning the gradual crystallization of the sample, evidenced by the steady continuous increase of the grain size up to 700 °C. However, the average grain size at this annealing remained below 20 nm, proving a true nanocrystalline state. Then, at 800 °C the sample was fully crystallized, with large grain sizes of over 100 nm. There was thus a sharp increase of the average grain size from the case of 700 °C annealing (16 nm) to the case of 800 °C annealing (more than 100 nm). Such a drastic increase corroborates the fact that the mechanisms of crystallization may have switched from the primary crystallization (governed by nucleation) to the secondary crystallization (governed by the accelerated growth of the grains). It is obvious then that at 800 °C, annealing the crystallization process has been completed. Observing the evolution of the lattice parameter with the annealing temperature reveals another interesting aspect. It is seen that the lattice parameter of the fcc Fe_3_Pt phase increased slightly from the as-cast case, and above the annealing temperature of 600 °C, it remained constant up to the maximum annealing temperature of 800 °C. This proves a remarkable stability of the lattice which, associated with the grain growth observed during annealing, hints toward a grain microstructure that is homogeneous, with lesser crystal defects, even at the highest annealing temperature where the coarsening of the grain size becomes evident. 

### 3.2. TEM Analysis

The above considerations about the microstructure gain even more support from the transmission electron microscopy analysis.

**Figure 3:**
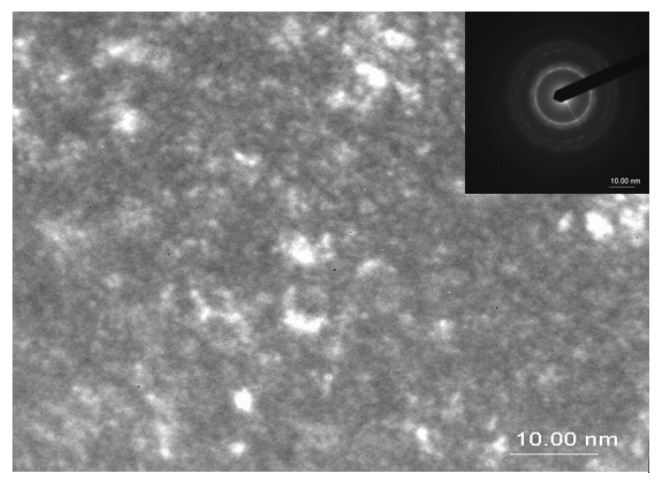
TEM image and corresponding EDP for the sample annealed at 500 °C.

**Figure 4:**
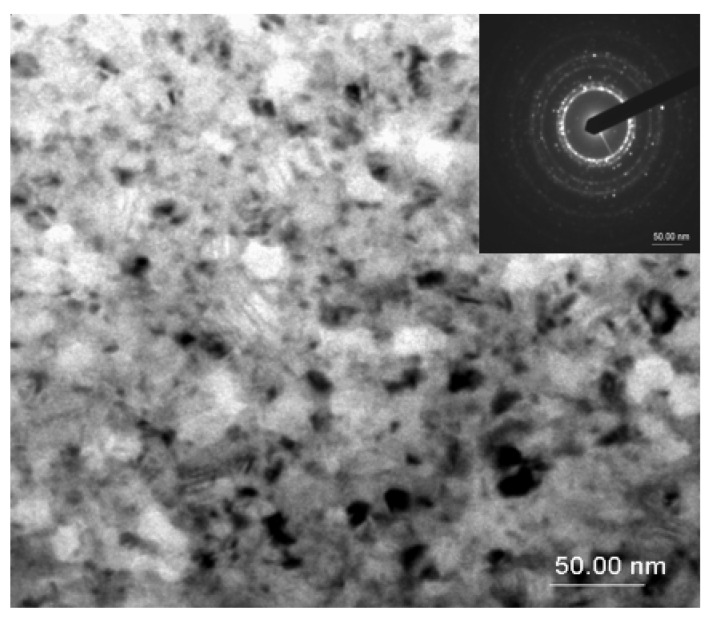
TEM image and corresponding EDP for the sample annealed at 700 °C.

**Figure 5:**
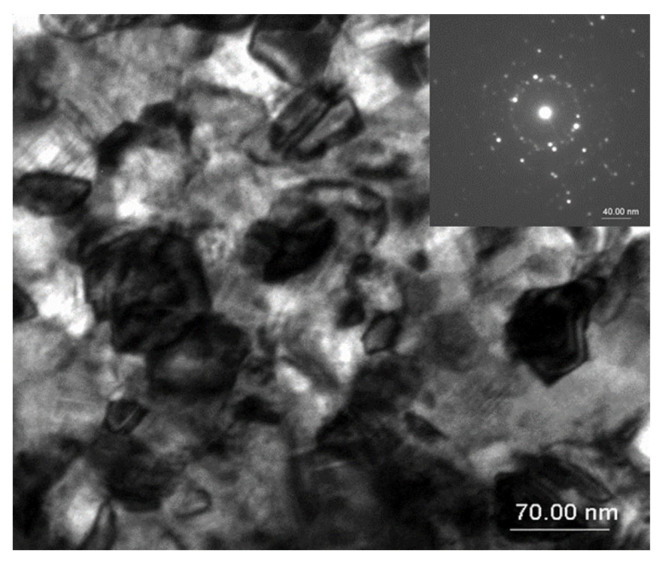
TEM image and corresponding EDP for the sample annealed at 800 °C.

In order to directly observe the microstructure obtained after annealing and the grain disposal within the samples, transmission electron microscopy images were taken for the samples annealed at 500 °C, 700 °C, and 800 °C. Figure 3, Figure 4 and Figure 5 show the bright field transmission electron microscopy image of the three annealed samples.

For the sample annealed at 500 °C, the TEM image presents features that are compatible with topologically disordered alloys. Few 3–5 nm nanocrystals are observable, and are dispersed within an amorphous-like matrix that gives lower contrast in the TEM image. The electron diffraction patterns (inset of Figure 3) show diffraction rings that are characteristic to the disordered fcc phase symmetry, in agreement with the XRD results. The grain size directly derived from TEM images is in remarkable agreement with those obtained from the numerical fittings of the XRD patterns.

For the sample annealed at 700 °C for 20 min (Figure 4), a microstructure consisting of well-formed, closely packed nanograins is clearly observable, the remaining amorphous matrix being less visible. The grains are larger than in the case of the sample annealed at 500 °C. Here, the electron diffraction pattern changes its features, and apart from the observed diffraction rings, several diffraction spots are observed. This proves the increase of the crystallite sizes and more localized electron diffraction, associated with the increase of the crystallographically coherent domain size in the fcc Fe_3_Pt.

The sample annealed at 800 °C (Figure 5) exhibits a microstructure where very large faceted grains (between 70 and 110 nm) can be seen, which are well-defined, almost regularly dispersed, and with different image contrasts. The contrast difference indicates the multiple phase character of the observed grains, as well as different crystallographic texturing.

The electron diffraction pattern shows multiple bright diffraction spots, characteristic to the polycrystalline state and only very weak diffraction rings, as was the case in the nanocrystalline state at 500 °C. As practically no more diffuse regions are observed, one may conclude that at 800 °C annealing, there were no more amorphous-like remaining matrices in the microstructure, the sample being fully crystallized. Taking into account the fact that only a small part of the microstructure is imaged by TEM and the results derived are highly local, the agreement with the grain size derived from the XRD (Table 1) is quite remarkable.

### 3.3. Mössbauer Analysis

**Figure 6:**
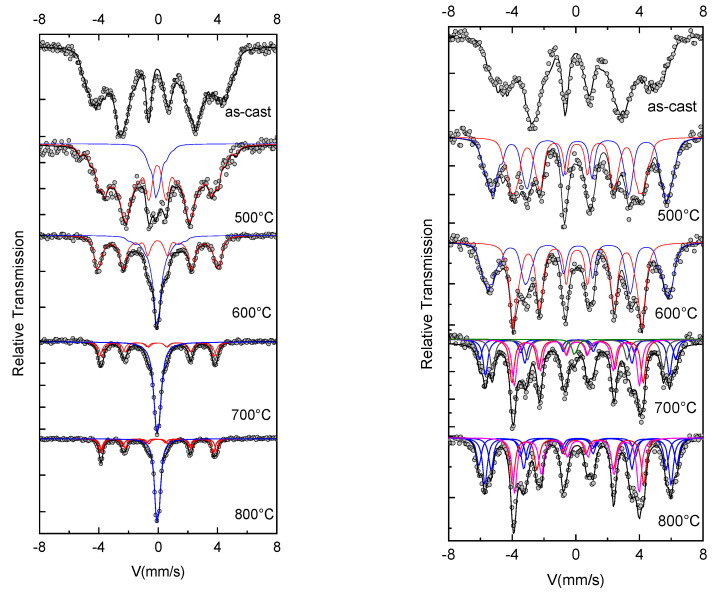
300 K and 77 K Mössbauer spectra of as-cast and annealed samples.

**Figure 7:**
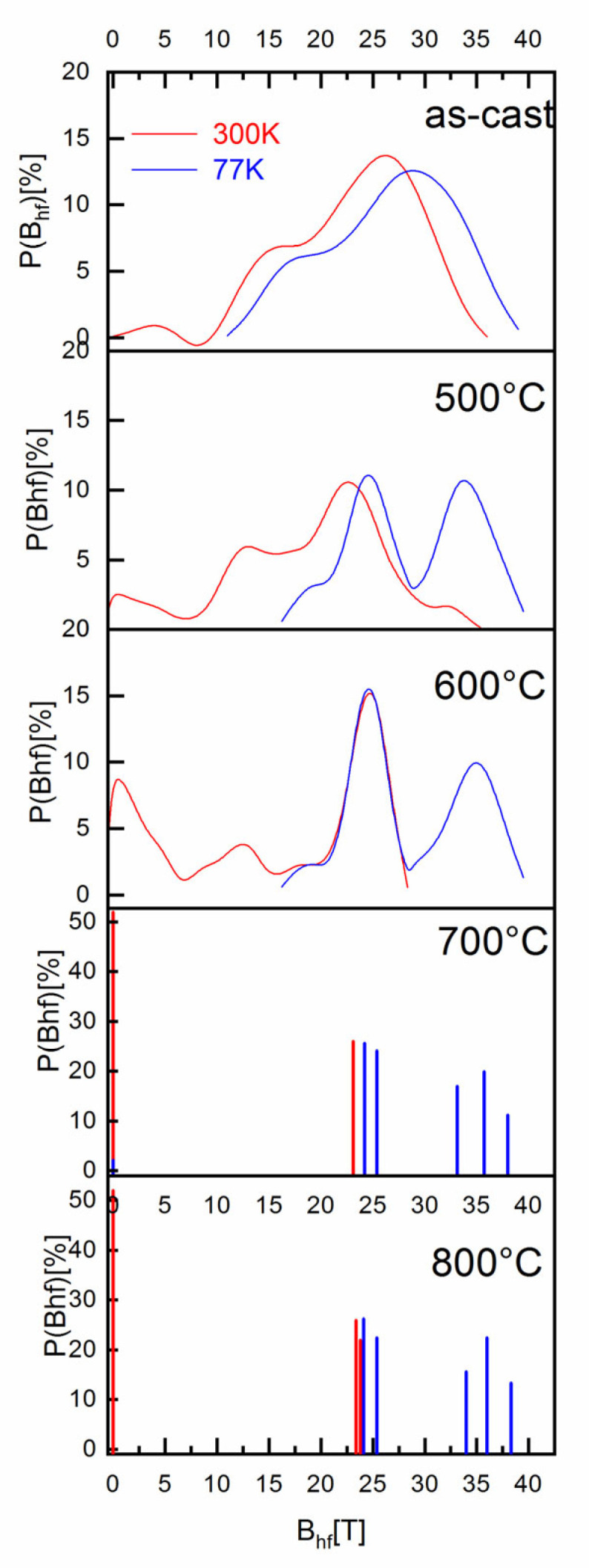
*B_hf_* distributions obtained for 300 K and 77 K spectra of samples annealed at 500–800°C for 20 min. The corresponding spectra are those depicted in Figure 6.

The next structural investigation of the as-cast and annealed samples was performed using ^57^Fe Mössbauer spectrometry. All Mössbauer spectra were analyzed using Mosfit software [19], a Lorentzian line-fitting program which allows the use of discrete distributions of hyperfine parameters corresponding to the different Fe chemical environments. Figure 6 is showing all the 300 K and 77 K Mössbauer spectra together with their fitting, showing individual contributions. The results of the fitting, meaning the obtained hyperfine parameters, as well as the identification of each individual contribution to the Mössbauer spectra, are given in Table 2. Figure 7 presents the as-obtained hyperfine field distributions for each of the Mössbauer spectra, as resulted from the fitting.

The Mössbauer spectrum for the as-cast sample (the top graphs) exhibits broad magnetic sextets, typical of distributed Fe environments encountered in Fe-rich amorphous ribbons. The shape of the spectra confirms the XRD results where an amorphous-like solid solution has been identified. The hyperfine field B_hf_ distributions derived from the fitting of the Mössbauer spectra show a bimodal-type large Gaussian profile distribution, which is characteristic for disordered Fe environment encountered in amorphous-like alloys, with two main chemical environments for Fe. From the numerical fitting of the size distribution with two Gaussian profiles, we have obtained for the low field mode an average B_hf_ of 14 T and 16 T for 300 K and 77 K, respectively, and for the high field mode an average B_hf_ of 26 T and 28 T for 300 K and 77 K. respectively. The relative proportion of the high-field to low-field relative contributions to the B_hf_ distributions is about 3:1. From the obtained B_hf_ values we can presume that the low field distribution corresponds to a disordered precursor phase, rich in boron, which would give rise upon annealing to a boride (possibly Fe_2_B) phase. On the contrary, the high field distribution is attributed to another disordered chemical environment, rich in iron, would supposedly promote, upon annealing, the formation of the fcc FePt grains. The observation of two different hyperfine field distributions hints to the potential formation of two different magnetic phases upon annealing.

The Mössbauer spectra of the annealed samples were also fitted with different discrete distributions of hyperfine parameters, corresponding to the different Fe chemical environments. A similar fitting model was used throughout the entire series of samples to ensure the reliability of the results. All the *B_hf_* distributions obtained for 300 K and 77 K spectra are plotted in Figure 7.

As explained earlier, the shape of the hyperfine field distribution of the as-cast sample reveals the existence of two separate iron environments which can be considered as incipient “nuclei” of the phases that will develop under thermal processing. As the annealing temperature increases, the process of forming two separate phases starts to be observed.

The shape of the spectra recorded at 300 K, for the sample annealed at 500 °C, exhibits a low spectral resolution, similar to that of the as-cast sample. By reducing the measuring temperature to 77 K, the spectral resolution allows us to distinguish two magnetic phases. Nevertheless, the Zeeman sextet lines associated with each phase are large enough, signifying the presence of a poorly ordered crystalline state. This is consistent with the amorphous-like features, the broad Bragg lines, observed in Figure 1. To take into account this crystalline state, the 77 K spectrum is refined using a discrete distribution of fields ranging from 5 to 40 T. Table 2 shows the mean values for the hyperfine parameters, as obtained from the fit.

The 500 °C spectrum also shows a broad line sextet, a pattern that is typical for distributed Fe environments, but the *B_hf_* distributions are narrower than in the as-cast state

At 77 K, Figure 7 shows a bimodal result of the hyperfine field distribution centered around 25 T and 34 T, both having almost the same abundance. Data from the literature [2,20,21,22,23] show that these hyperfine field values can be assigned to the iron boride Fe_2_B phase and the fcc FePt (A1) phase, respectively. At 300 K, the *B_hf_* distributions exhibits a similar form as observed for the as-cast sample, the best fit was obtained with two subcomponents. One magnetic component (red line in Figure 6) has large peaks, typical for disordered Fe environments, is predominant, and has a relative intensity of 89%. It is attributed to the Fe-B rich amorphous-like phase, but exhibits an average hyperfine field value lower than that of Fe_2_B. The second, at low field (blue line in Figure 6) of about 11% with an average *B_hf_* value of around 2 T, is attributed to a highly disordered fcc A1 phase having small sizes (estimated at 7 ± 2 nm, see Table 1). The Fe_2_B phase is reportedly stable at high temperatures [24] and is characterized by two magnetic sextets with hyperfine fields of about 23.3 and 24.2 T at 300 K.

The appearance at 77 K of a second pic at around 34 T, identified as the fcc Fe_3_Pt phase, is the result of the effect of thermal energy on the magnetization dynamics. Indeed, the XRD results at ambient temperature clearly show the presence of the fcc Fe_3_Pt phase, however this was not evident on the Mössbauer spectra recorded at 300 K.

In fact, C.B. Rong et al. [25] studied the dependence of Curie temperature on chemical composition of annealed Fe_x_Pt_100-x_ nanoparticles. They showed that for 70 < x < 80, T_C_ is between 600 K and 200 K. This may explain the differences between the magnetic features of the same fcc Fe_3_Pt phase: sextet at 77 K and singlet at 300 K.

The shape of the Mössbauer spectra at 600 °C supports the emerging bimodal microstructure observed at 500 °C. In fact, the spectral width of the Mössbauer component lines is here narrower, marking a better crystalline state of the grains with increasing average size, under the influence of the thermal processing. This is consistent with XRD results displayed in Figure 1 and Table 1.

The 300 K spectrum clearly displays two phases: one magnetic phase with an average hyperfine field value of about 21 T, and a slightly magnetic central peak with a hyperfine field value of around 2 T. Compared with the 500 °C sample, changes in the abundance of the two sub-components show progressive growth of the fcc Fe_3_Pt phase to the detriment of the remaining Fe–B rich amorphous-like phase.

The spectrum and *B_hf_* distribution at 77 K confirm the previous indications of a paramagnetic behavior of fcc Fe_3_Pt grains, about 10 nm in diameter (see Table 1). This para-ferromagnetic transition of the fcc A1 Fe_3_Pt is preserved also for the higher annealing temperatures.

The 300 K spectra for the samples annealed at 700 °C and 800 °C show features similar to the 600 °C spectrum. It consists of sextets attributed to the Fe_2_B and paramagnetic component, assigned to the superparamagnetic fcc Fe_3_Pt nanograins. The *B_hf_* distribution is narrower, as can be observed in Figure 7, and the individual linewidths are also narrower (see Table 2) than those of the 600 °C annealing. The fct Fe_2_B contribution becomes here well deconvoluted into the two magnetic sextets, usually encountered in the literature for Fe_2_B, with *B_hf_* of 24.2 T and 23.1 T, respectively. The paramagnetic blue component becomes predominant in the sample, reaching more than 50% relative abundance. It shall be noted that, in the diffraction studies, the Bragg lines of Fe_2_B are of extremely small intensity, while in Mössbauer analysis the relative proportion of Fe_2_B is found to be higher. This can be explained by the high structure factor of Pt that, in X-ray diffractograms, hinders the occurrence of Fe_2_B Bragg lines.

The 77 K spectra for samples annealed at 700 °C and 800 °C are highly similar: the same fitting model is used and the same components are retrieved, as in the 600 °C case. This feature is also observed in the *B_hf_* distributions plotted in Figure 7. The HF distributions are narrower, signaling the continuous process of structural refinement, as proven also by the XRD results. While at 700 °C annealing, Fe_2_B and Fe_3_Pt have almost the same relative abundance of 50%, at 800 °C annealing the Fe_3_Pt predominates having a relative abundance of 52%. A small fraction of A1 fcc FePt remains still magnetically disordered as shown by the paramagnetic component of about 2% relative abundance, even after annealing at 800 °C.

The Mössbauer spectra confirm thus the XRD results where we have observed the formation of cubic Fe_3_Pt phases and Fe_2_B starting with the annealing at 700 °C. However, the gradual phase evolution shows various paths for achieving this two-phase magnetic structure, depending on the annealing temperature chosen.

To summarize the phase evolution that has been observed in the Fe_68_Pt_13_Nb_2_B_17_ sample, as documented by the XRD and Mössbauer results, the following sequences of crystallization, with main phases observed, may be depicted as in the scheme:

1. 500 °C–600 °C: Amorphous-like fcc *A*1 disordered → fcc Fe_3_Pt + FeB-rich + residual

2. 700 °C–800 °C: fcc Fe_3_Pt + FeB-rich + residual → fcc Fe_3_Pt + fct Fe_2_B

It is also shown that in the presence of additional magnetic phases, such as Fe_2_B, the fcc FePt undergo a chemical environment transition from magnetically ordered at 77 K to magnetically disordered at 300 K.

In the following we will undertake temperature dependent investigations in order to observe the dynamics of this chemical environment transition of the Fe_3_Pt phase, by measuring Mössbauer spectra at several temperatures, ranging from 300 K down to 4.2 K. This study will then be accompanied by extensive temperature-dependent magnetic measurements.

### 3.4. Magnetic Phase Evolution

**Figure 8:**
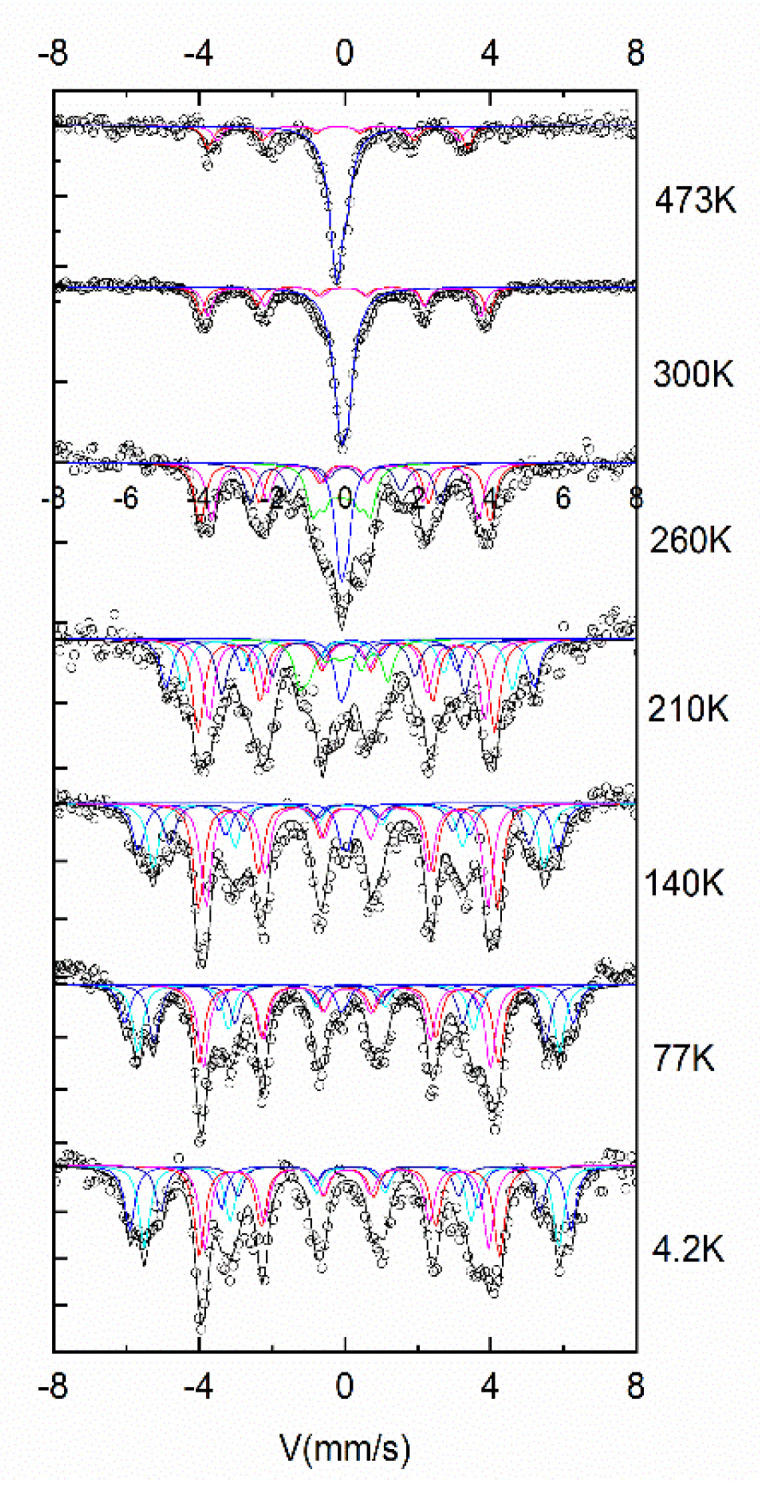
^57^Fe Mössbauer spectra recorded at various temperatures for the sample annealed at 700 °C.

In order to properly monitor the evolution with temperature of the magnetic structure of the samples, we have undertaken two different experiments. In the first experiment, we have taken Mössbauer spectra at various temperatures, in an extended range, from 473 K (200 °C) down to 4.2 K. Figure 8 presents the recorded Mössbauer spectra at various temperatures, together with the fitting of their constituent magnetic phases.

**Figure 9:**
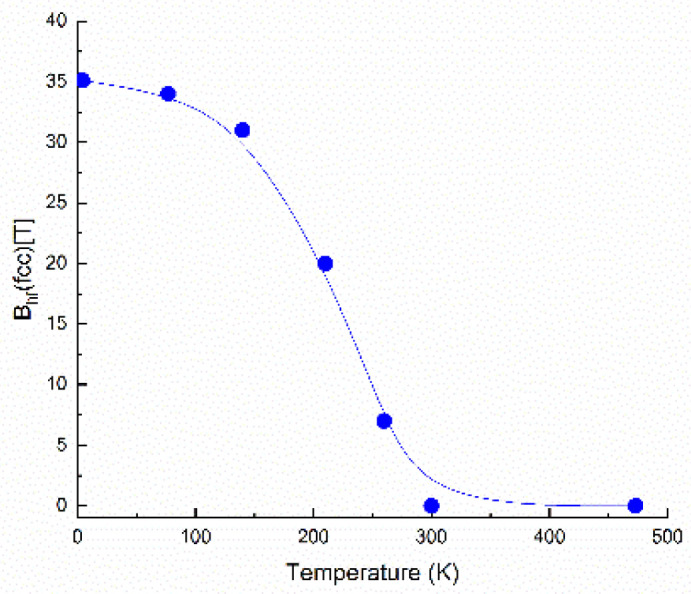

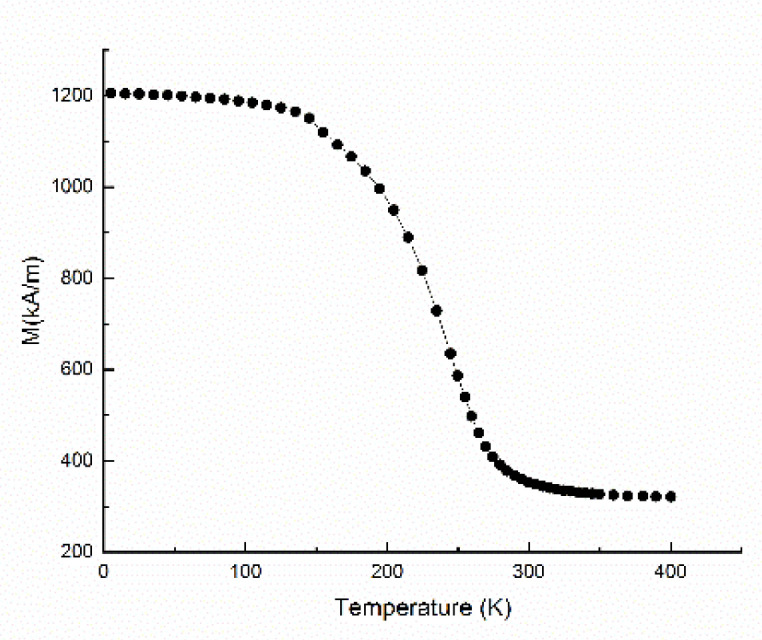
(**top**): B_hf_ vs. T for Fe_3_Pt, as resulted from the fitting of Figure 8 spectra; (**bottom**): magnetization variation with temperature M(T) recorded using VSM.

It can be observed that at 473 K and 300 K the spectra are quite similar, being composed of two main components: the paramagnetic (singlet) component, assigned to fcc FePt (blue color in the Figure 8), and the ferromagnetic (sextet) component, assigned to tetragonal Fe_2_B (red color in the Figure 8). However, below room temperature, starting with 260 K, part of the paramagnetic component starts to become magnetically split, and the spectrum becomes multicomponent. The transformation from paramagnet to ferromagnet with the decrease in temperature is gradual, however at about 100 K, the transformation is almost complete. At 4.2 K the FePt phase appears fully ferromagnetic. This evolution with temperature is more visible if one plots (Figure 9, top) the average hyperfine field, as resulted from the fitting of Mössbauer spectra in Figure 8, as a function of temperature. It can be seen that going down in temperature below 300 K makes the average hyperfine field increase quite sharply, between 260 K and 77 K, and attaining saturation below 77 K, reaching up to about 35 T, value that is reached at 4.2 K. This value is consistent with hyperfine field values usually encountered in Fe-rich alloys.

The second experiment, aiming at monitoring the evolution with temperature of the magnetic properties of the sample annealed at 700 °C, was made by recording the magnetization as a function of temperature in an experiment performed using the vibrating sample magnetometer in an applied field of 0.3 T. The graph depicting the magnetization vs. temperature M(T) is plotted in Figure 9 (bottom). The measurement protocol employed consisted of heating the sample up to 400 K and starting measurement of the specific magnetization at 400 K while the system is steadily cooled down to 4.2 K with a continuous cooling rate of 2 K/min and under an applied field of 0.3 T. It can be observed that at 400 K the sample exhibits low specific magnetization (about 200 kA/m) and this value remains almost constant up to about 300 K where the magnetization starts to increase. Between 270 K and down to around 150 K there is a sharp and steady increase of the specific magnetization up to about 1100 kA/m. From about 120 K the magnetization stabilizes up to a saturation value of about 1200 kA/m. This situation is explainable by taking into account the phase structure at every temperature. At high temperatures, the fcc Fe_3_Pt is in its paramagnetic state, as proven by the Mössbauer results, therefore its contribution to the total magnetization of the sample is null. The value recorded of around 200 kA/m represents only the contribution of the fct Fe_2_B phase. Below 270 K, the magnetization starts to increase, as the Fe_3_Pt phase starts having non-zero contributions to the total magnetization of the sample, due to the gradual transition para to ferro observed in Figure 9. The sharp increase of the magnetization, upon decreasing the temperature from 270 K to 120 K, illustrates thus the para to ferro magnetic transition. This transition is proven to be gradual, as it happens over an interval of over 150 K. Since at lower temperatures, the total magnetization saturates at about 1200 kA/m, and at higher temperatures the minimum value (given only by the contribution of Fe_2_B, Fe_3_Pt being paramagnetic) is 200 kA/m, we can conclude that roughly the maximum contribution to the total magnetization at the lowest temperature investigated in the measurement is about 1000 kA/m, a value in very good agreement with the values reported in the literature for the Fe–Pt binary magnetic phases. This behavior is in very good agreement with the average hyperfine field dependence of temperature B_hf_(T), where we have also seen a gradual increase of B_hf_ from 260 K down to about 100 K, exactly like in the case of M(T).

This temperature dependence of the magnetization measurement has allowed us to confirm once again the chemical environment transition of the fcc Fe_3_Pt from the paramagnetic state at 300 K and above, to the ferromagnetic state at lower temperatures (120 K and below). Moreover, we have proven that this transition occurs gradually, over a large temperature interval of about 150 K. This transition was surprisingly observed only through Mössbauer studies. It is clear that the overall magnetic properties recorded at 300 K of the sample annealed at 700 °C where Fe_3_Pt and Fe_2_B co-exist, i.e., coercivity and saturation magnetization are actually dictated by the Fe_2_B, as the Fe_3_Pt is paramagnetic at 300 K. Moreover, the Curie temperature of Fe_3_Pt [25] was shown to decrease strongly from 550 K to 310 K when Fe content varied with only 3% around the stoichiometric value for Fe_3_Pt. This strong deviation can be explained by changes in Fe_3_Pt atom ordering, where lower Fe content may produce a tetragonal ordering of L1_0_ type, with a higher T_C_ rather than an ordering of L1_2_ type, typical for Fe_3_Pt, which has a lower T_C_. This temperature variability of the magnetic ordering has been also evidenced in some recent papers. A temperature-dependent study of the anisotropy field of L1_0_ FePt near T_C_ [13] showed that the scaling law (1 – *T*/*T*_c_)^β^ holds experimentally only for β values close to 0.36 and thermally activated magnetization reversal at temperatures near T_c_ cannot be ignored, even at time scales smaller than 1 ns. Other kinds of incoherent magnetic switching have been evidenced in equiatomic L1_0_ FePt grains [14]. In addition, on thin films of FePt with variable concentrations oof Fe [15], it was found that magnetic ordering was highly dependent on the actual composition and the temperature.

**Figure 10:**
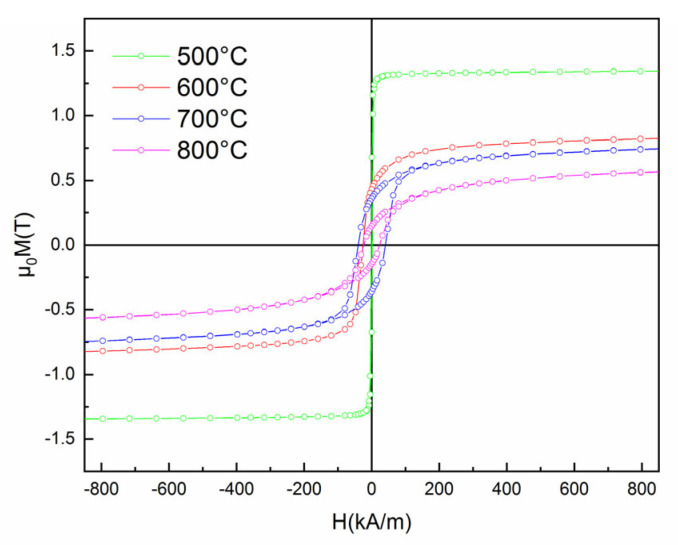
The hysteresis loops for the samples annealed at various temperatures.

Further magnetic properties investigations of the samples annealed for 20 min at various temperatures were carried out by measuring the major hysteresis loops, which were recorded at 300 K using a VSM, under a parallel applied field of up to 1.5 T. The obtained hysteresis loops are presented in Figure 10.

**Figure 11:**
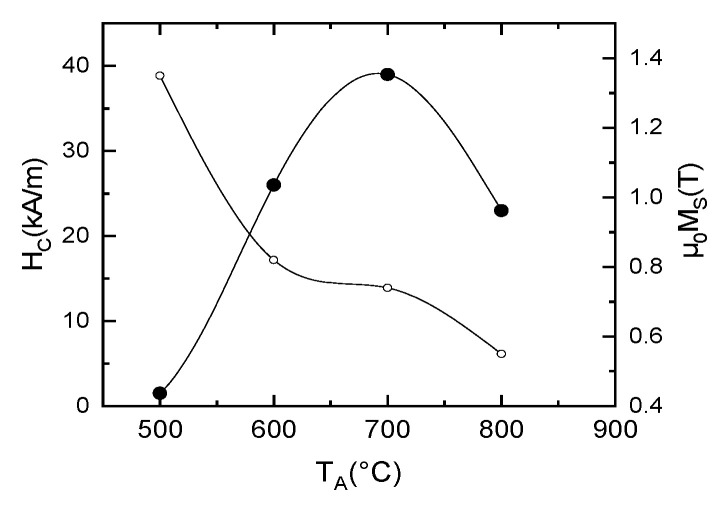
Coercive fields and saturation magnetization values obtained for the annealed samples, as a function of annealing temperature.

The curve for the sample annealed at 500 °C is typical for soft magnetic materials, with virtually no coercivity, a fast approach to saturation, and high saturation magnetization (cca. 1.3 T), but as the annealing temperature goes up, the saturation magnetization diminishes due to the continuous increase of the grain size and the steady increase of the paramagnetic contribution observed in the Mössbauer spectra. Most interesting is that a magnetic hardening occurs in the annealed sample, with non-negligible coercive fields recorded for the annealed samples. The coercivity reaches a maximum for the sample annealed at 700 °C. All the values of the coercive fields and of the saturation magnetization recorded in the hysteresis loops are plotted as a function of annealing temperature in Figure 11. It can be observed that the coercivity reaches indeed a maximum for the annealing at 700 °C which is now confirmed to be the optimal annealing temperature for improving the magnetic properties of the alloy. As for the saturation magnetization, these values decrease slowly with an increase in the annealing temperature, from around 1.4 T for 500 °C annealing down to about 0.5 T for the 800 °C annealing. Such behavior of the coercivity is explainable by the phase evolution of the samples during annealing. As the metastable as-cast state is magnetically soft, upon annealing, the crystals nucleate and grow in a tridimensional model. The formed nanograins are initially monodomains, for intermediate stages of annealing 500 °C–600 °C. After annealing at 700 °C, the average grain size is only 16 nm. This refined microstructure is formed of grains from two magnetic phases and due to various reversal mechanisms, the coercive field increases up to the maximum. After 800 °C annealing, the grains have furthermore increased up to 100 nm (see Table 1) and this leads to the decrease of the coercivity as observed in Figure 11. This is hereafter interpreted in terms of a log H_c_ vs. log D dependence that resembles partly with a previously developed random anisotropy model.

The random anisotropy model, initially proposed by Alben et al. [26] and reformulated by Herzer [27,28], proposes to explain the dependence of various grain size-dependent magnetic properties through the interplay of exchange coupling between grains and magnetic anisotropy energy. The distributed grain microstructure leads to a distribution of magnetic easy axes with various orientations over the scale of the structural correlation length which is here assimilated to the average grain size *D*. The model of scaling these dependences, the random anisotropy model, is based on the exchange correlation length *L*_0_, the length from which the exchange coupling overcomes the anisotropy energy barrier:

(1) $$L_{0}=\varphi \sqrt{A/\left|K_{1}\right|}$$

where *A* is the exchange stiffness, $\left|K_{1}\right|$ is the local magnetic anisotropy constant, and *ϕ* is a proportionality factor, here assumed to be one. The random anisotropy model shows that for nanocrystalline materials with distributed grain size at the nanometric scale, there is a scaling formula linking the average anisotropy $\langle K_{1}\rangle$ to the local anisotropy $\left|K_{1}\right|$:

(2) $$\langle K_{1}\rangle =\left|K_{1}\right|{\left(D/L_{0}\right)}^{6}$$

The coercive field is linked to the average magnetic anisotropy through the formula:

(3) $$H_{c}=p_{c}\frac{\langle K_{1}\rangle }{J_{s}}$$

where p_c_ is a proportional constant and J_s_ is the saturation magnetization [20].

Some typical *L*_0_ values for Fe-based alloys are between 20–40 nm for Fe-based alloys. Therefore, for most amorphous and nanocrystalline materials, including our samples annealed at 500 °C until 700 °C, we are in the situation that D < L_0_. Combining Equations (2) and (3) we observe that the coercive field is scaling with D^6^, for D < L_0_ cases where the exchange anisotropy energy exceeds the exchange coupling.

**Figure 12:**
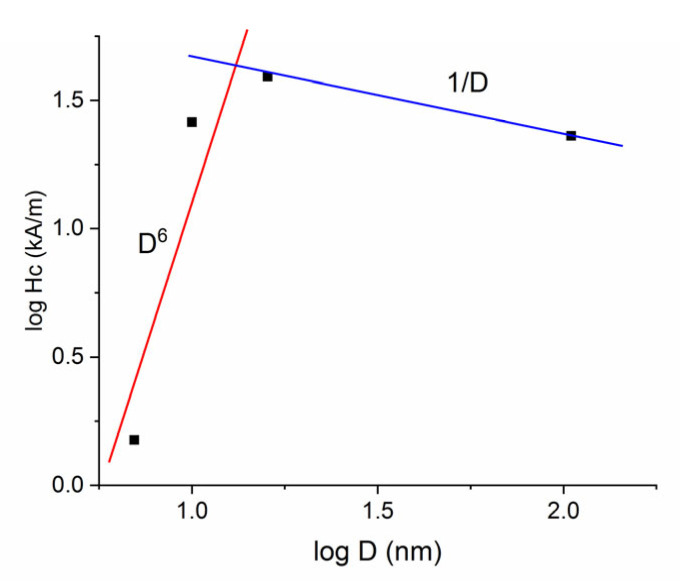
Average grain size dependence of the coercive field. Full symbols are the experimental values from Figure 11 plotted versus the average grain size in logarithmic scale. The linear fitting gives a D^6^ dependence (red line) for samples annealed between 500 °C and 700 °C and a 1/D dependence (blue line) for annealing between 700 °C and 800 °C.

The grain size dependence of the coercivity is displayed in Figure 12, where we have plotted the coercive field values as revealed in Figure 11 versus the average grain sizes D, as calculated in Table 1. There are two dependences observed and linearly fitted in the graph. For the samples annealed between 500 °C and 700 °C the coercivity values follow a D^6^ dependence (red line) while for annealed temperatures between 700 °C and 800 °C, due to the sharp increase of the grain sizes, the coercive field decreases and follows a 1/D dependence (blue line). This result presents similarities with the random anisotropy model for the samples annealed at 500 °C and shows good resemblance to other coercive field dependences on grain size, as reported previously in other papers [29,30,31]. It must however be mentioned that the random anisotropy model is applicable mostly to amorphous and nanocrystalline alloys, having less relevance to multiple-phase polycrystalline systems, as is the case for instance for the sample annealed at 800 °C.

The magnetic behavior is consistent with a microstructure made of small grains of multiple magnetic phases with randomly distributed easy axes and with small coupling energy, as revealed in other materials with multiple magnetic phases [32], pointing to a reduced exchange coupling between adjacent magnetic grains. 

## 4. Conclusions

By using the rapid solidification from the melt, an alloy from the Fe–Pt–Nb–B system has been synthesized in order to investigate the formation of multiple magnetic phases and to monitor their interplay with respect to the magnetic performances. It has been shown that the annealing at 500 °C to 600 °C promotes formation of two main metastable nanocrystalline phases: fcc A1 (FePt-based) and FeB-rich phase in the primary crystallization stage, dominated by nucleation of crystallites. Further annealing at 700 °C and 800 °C promotes formation of fcc Fe_3_Pt and fct Fe_2_B crystalline phases in the secondary crystallization stage, dominated by grain growth processes and poly-crystals formation. Complex ^57^Fe Mössbauer spectroscopy studies have allowed precise identification of the constituent phases as well as determination of hyperfine parameters of each phase in every stage of annealing. A surprising para to ferromagnetic transition was observed for the fcc A1 disordered FePt-based phase, this phase being proven to be paramagnetic at 300 K and ferromagnetic at 77 K. Moreover, by temperature-dependent ^57^Fe Mössbauer spectroscopy, we have shown that this transition is gradual, occurring over an interval of 100 K. The coercive field evolution upon annealing has been successfully shown to validate the random anisotropy model and a D^6^ dependence of the coercive field was found for the primary crystallization nucleation-dominated stage. Moreover, a 1/D dependence of the coercive field was proven for the secondary crystallization growth-dominated stage. These results show potential to stimulate further interest in investigating two-phase magnetic materials exhibiting chemical environment magnetic transitions in well-defined temperature intervals, as well as to further use in applications where different magnetic states are required at different operating temperatures. 

## Figures and Tables

**Table 1:** The lattice parameters and the average grain sizes derived from the fit of the XRD spectra.

Sample	Annealing	*fcc* (Fe_3_Pt)	*fct* Fe_2_B		Grain Size Fe_3_Pt (nm)	Grain Size Fe_2_B (nm)
		a (Å)	a (Å)	c (Å)		
Fe_68_Pt_13_Nb_2_B_17_	As-cast	3.69			3 ± 1	
	500 °C_20′	3.72			7 ± 2	
	600 °C_20′	3.73			10 ± 2	
	700 °C_20′	3.73	5.11	4.25	16 ± 3	15 ± 3
	800 °C_20′	3.73	5.12	4.24	105 ± 7	40 ± 4

**Table 2:** Hyperfine parameters obtained from the fitting of the 300 K and 77 K Mössbauer spectra. δ is the isomer shift, Γ/2 is the half width at half maximum (HWHM) of the line, 2ε is the quadrupolar splitting, and Bhf is the hyperfine field.

	300 K						77 K					
	δ (mm/s)	Γ/2 (mm/s)	2ε (mm/s)	B_hf_ (T)	%	Phase	δ (mm/s)	Γ/2 (mm/s)	2ε (mm/s)	B_hf_ (T)	%	Phase
As-cast	0.15	0.18	0	23.5	100	am-like	0.25	0.18	0	27	100	am-like
500°C	0.16	0.18	0	21.2	89	Fe-B rich am-like	0.34	0.18	0.07	34	48	fcc Fe_3_Pt
	0.06	0.18	0.13	0.2	11	P* fcc Fe_3_Pt	0.24	0.18	−0.01	24	48	Fe-B-rich am-like
							0.33	0.18	0.65	0	4	Residual am-like
600°C	0.12	0.18	0.03	21	65	Fe-B rich am-like	0.33	0.18	0.03	34.3	45	fcc Fe_3_Pt
	0.09	0.30	0.02	0.2	32	P* fcc Fe_3_Pt	0.24	0.18	0.04	22.0	51	Fe-B-rich am-like
							0.24	0.18	0.6	0	4	Residual am-like
700°C	0.10	0.19	0.08	24.2	24	Fe_2_B	0.23	0.18	0.18	25.4	24	fct Fe_2_B
	0.13	0.19	−0.02	23.1	25	Fe_2_B	0.21	0.18	−0.05	24.2	26	fct Fe_2_B
	0.10	0.25	0	0	51	P* fcc Fe_3_Pt	0.26	0.18	0.00	38.0	11	fcc Fe_3_Pt
							0.28	0.18	0.00	35.7	20	
							0.26	0.18	0.03	33.1	17	
							0.07	0.18	0.00	0	2	
800°C	0.11	0.14	0.22	23.7	22	fct Fe_2_B	0.20	0.18	0.17	25.4	22	fct Fe_2_B
	0.11	0.14	−0.09	23.2	27	fct Fe_2_B	0.24	0.18	−0.05	24.1	26	fct Fe_2_B
	0.10	0.16	0.13	0	51	P* fcc Fe_3_Pt	0.28	0.26	0.00	38.3	13	fcc Fe_3_Pt
							0.28	0.18	0.00	36.1	22	
							0.31	0.18	0.03	33.9	16	

The estimated errors are: ±0.02 mm/s for IS and QS/2ε, ±0.1 T for B_hf_, and ±1 for the relative proportion. P*-paramagnetic.

## Data Availability

The data are not publicly available due to IPR protection measures. Access to part of the data can be granted on a case by case basis, upon request.
